# Assessment of blood cultures and antibiotic susceptibility testing for bacterial sepsis diagnosis and utilization of results by clinicians in Benin: A qualitative study

**DOI:** 10.3389/fpubh.2022.1088590

**Published:** 2023-01-16

**Authors:** Brice Boris Legba, Victorien Dougnon, Hornel Koudokpon, Sointu Mero, Riku Elovainio, Matti Parry, Honoré Bankole, Kaisa Haukka

**Affiliations:** ^1^Research Unit in Applied Microbiology and Pharmacology of Natural Substances, Research Laboratory in Applied Biology, Polytechnic School of Abomey-Calavi, University of Abomey-Calavi, Abomey-Calavi, Benin; ^2^Human Microbiome Research Program, Faculty of Medicine, University of Helsinki, Helsinki, Finland; ^3^Physicians for Social Responsibility, Helsinki, Finland; ^4^Tampere Center for Child, Adolescent, and Maternal Health Research (TAMCAM): Global Health Group, University of Tampere, Tampere, Finland; ^5^New Children's Hospital, University of Helsinki, Helsinki, Finland; ^6^Department of Microbiology, University of Helsinki, Helsinki, Finland

**Keywords:** sepsis, blood culture, antibiotic susceptibility testing (AST), antibiotic prescribing, Benin

## Abstract

**Objectives:**

We assessed the current status of blood culture and antibiotic susceptibility testing (AST) practices in clinical laboratories in Benin, and how the laboratory results are used by physicians to prescribe antibiotics.

**Methods:**

The qualitative study covered twenty-five clinical laboratories with a bacteriology unit and associated hospitals and pharmacies. Altogether 159 laboratory staff, physicians and pharmacists were interviewed about their perceptions of the state of laboratory diagnostics related to sepsis and the use of antibiotics. Face-to-face interviews based on structured questionnaires were supported by direct observations when visiting five laboratories in across the country.

**Results:**

Only 6 laboratories (24%) conducted blood cultures, half of them with a maximum of 10 samples per month. The most common gram-negative bacteria isolated from blood cultures were: *Escherichia coli, Salmonella* spp. and *Salmonella enterica* serovar Typhi while the most common gram-positives were *Enterococcus* spp. and *Staphylococcus aureus*. None of the laboratories listed *Klebsiella pneumoniae* among the three most common bacteria isolated from blood cultures, although other evidence indicates that it is the most common cause of sepsis in Benin. Due to limited testing capacity, physicians most commonly use empirical antibiotic therapy.

**Conclusions:**

More resources are needed to develop laboratory testing capacity, technical skills in bacterial identification, AST, quality assurance, and communication of results must be strengthened.

## Introduction

Microorganisms entering the bloodstream can trigger sepsis, which is the body's generalized response to an infection and a life-threatening condition. Sepsis is the third most common cause of death for children under the age of five (1). It is the most common cause of hospital deaths and the leading cause of neonatal mortality, particularly in low- and lower-middle-income countries (LMICs) (2–4). Its incidence depends on complex interplay between factors related to the host, pathogen and health system response (5). Several chronic diseases, sociodemographic factors, poor access to health care systems and quality of care are associated with the occurrence of sepsis and its case fatality rate (6).

Sepsis is most commonly caused by aerobic bacteria (7). In LMICs the prevalence data are notably limited by the restricted ability to culture and identify organisms using standard microbiological techniques. In addition to the pathogenicity of bacterial strains, the major concern is their increasingly common resistance to the antibiotics used in the treatment of sepsis (8). For example, according to the World Health Organization (WHO), resistance of *Klebsiella pneumoniae*, the major cause of bloodstream infections, to the carbapenem antibiotics used as last-resort treatment has spread to all regions of the world (9). Antimicrobial resistance (AMR) is in fact one of the major challenges in the management of sepsis, in particular in LMICs (4, 10).

The bacteriology laboratory has twofold strategic role in the diagnosis of sepsis. Firstly, at the individual level, laboratory tests such as a blood culture and antibiotic susceptibility testing (AST) confirm the clinical diagnosis by identifying the causative organism and providing data on the susceptibility of the organism to antibiotics (11). Secondly, for the clinic, laboratory diagnostics provide relevant local information as a basis for the empirical use of antibiotics. Through both mechanisms, the test results contribute toward prescribing the appropriate antibiotic for the effective treatment of sepsis. Optimal testing activity and utilization of results requires close collaboration between laboratory staff, physicians and pharmacists, who provide antibiotics to the patients based on the prescriptions by physicians (12).

In Benin, laboratory diagnostics of clinical conditions such as sepsis is very limited. As in sub-Saharan Africa in general, clinical laboratories are typically poorly linked to clinical services, insufficiently resourced, and, therefore, under-utilized (13, 14). Furthermore, access to laboratory tests and drugs depends on capacity of the patients to pay for them, which often leads to tests not being done, and appropriate antibiotics not purchased (15). There is no national surveillance data for sepsis in Benin. However, in a recent study in central Benin, antibiotic resistant pathogens such as *K. pneumoniae, Salmonella enterica* serovar Typhi and *Staphylococcus aureus* were isolated from blood cultures (14). Studies on other clinically important pathogenic bacteria show AMR to be common in Benin (16, 17).

We wanted to find out the number and performance level of Beninese clinical laboratories that conduct microbiological tests required for the management of sepsis, especially blood cultures and AST, across the country. We also studied whether physicians make sufficient use of laboratory results when prescribing antibiotics and whether pharmacists have sufficient knowledge when dispensing antibiotics when there is a limited selection. We therefore conducted a qualitative study to investigate understanding, perceptions, knowledge, skills and practices of these three groups of professionals. We also studied resources available to them and their professional development needs. We conducted face-to-face interviews using structured questionnaires with all three groups and supplemented the results with discussions and visits to selected clinical laboratories. The results will be used to develop appropriate policies for antibiotic prescription and stewardship, to improve sepsis management and to strengthen diagnostics and treatment of infectious diseases in general.

## Methods

### Study setting, sample size and inclusion criteria

The study was conducted in Benin in West Africa. We wanted to involve all laboratories from different parts of the country with a functional bacteriology unit, therefore we contacted all the potential health centers and other stakeholders, such as the Association of Medical Biologists of Benin. There are almost 150 authorized clinical diagnostic laboratories in Benin. An initial survey identified all those that conduct bacteriological tests, including blood culture and/or AST and we subsequently identified 27 such authorized laboratories. Two laboratories failed to respond to our request; thus, the study was carried out with 25 laboratories. The laboratories were at different levels of the healthcare system: 2 from the central or national level, 4 from the intermediate or departmental level (Departmental Hospital Centers); and 19 from the peripheral level (Zone hospitals (HZs), health centers, unattached pharmacy dispensaries (independent pharmacies, i.e., not integrated or connected to a hospital or health center), unattached maternity wards (health facilities providing only a maternity services, i.e., the maternity unit is not integrated or connected to a hospital or health center and faith-based health centers).

All but one laboratory were affiliated with a healthcare facility. Ten facilities were private, 9 public and 6 faith-based hospitals located in different parts of the country. At each site, we aimed to interview a laboratory technician, a laboratory manager, three physicians and two pharmacists. We interviewed technicians who conduct AST and/or blood culture on a daily basis. The laboratory managers were in charge of the laboratory. The physicians and pharmacists were practicing, not administrative, staff. The physicians were working in the general medicine service, intensive care, pediatrics or emergency medicine. Physicians sending patients to the non-hospital connected private laboratory were identified in the nearby hospitals for the interview. Several of the healthcare facilities did not have an in-house pharmacy or the in-house pharmacy had only one pharmacist. In these cases, pharmacists from pharmacies located close to the facility or a pharmacy most frequently visited by patients were included in the study. These private pharmacies were identified based on the recommendation of the interviewed physicians.

In total, we interviewed 25 laboratory technicians, 25 laboratory managers, 62 physicians and 47 pharmacists. Before data collection, informed consent was obtained from all participants. The interviews were conducted anonymously.

### Data collection and analysis

The data was collected between June 7 and June 19, 2021. We used structured on-site, face-to-face interviews using tablets with a KoBoToolbox application (www.kobotoolbox.org/). The digitized data collection allowed for online data transfer and real-time quality control by a core team in Cotonou checking all forms and correcting the missing or inaccurate information immediately.

The data collected included general information about the hospital/laboratory and on the participating professionals. From the laboratory staff, we collected data on the practices related to blood cultures and AST. The questions were largely based on the WHO standard procedures (18). Physicians were interviewed about their practices of requesting a blood culture and AST before prescribing antibiotics and the interpretation of AST results. Pharmacists were asked about their knowledge and practice in dispensing antibiotics for the treatment of sepsis.

Data analysis was performed using SPSS version 24. The analysis was essentially descriptive and qualitative. A content analysis was carried out regarding the goals related to the identification of capacity development needs aimed at improving the blood culture and AST practices.

## Results

### Study sites and background information

**Figure 1:**
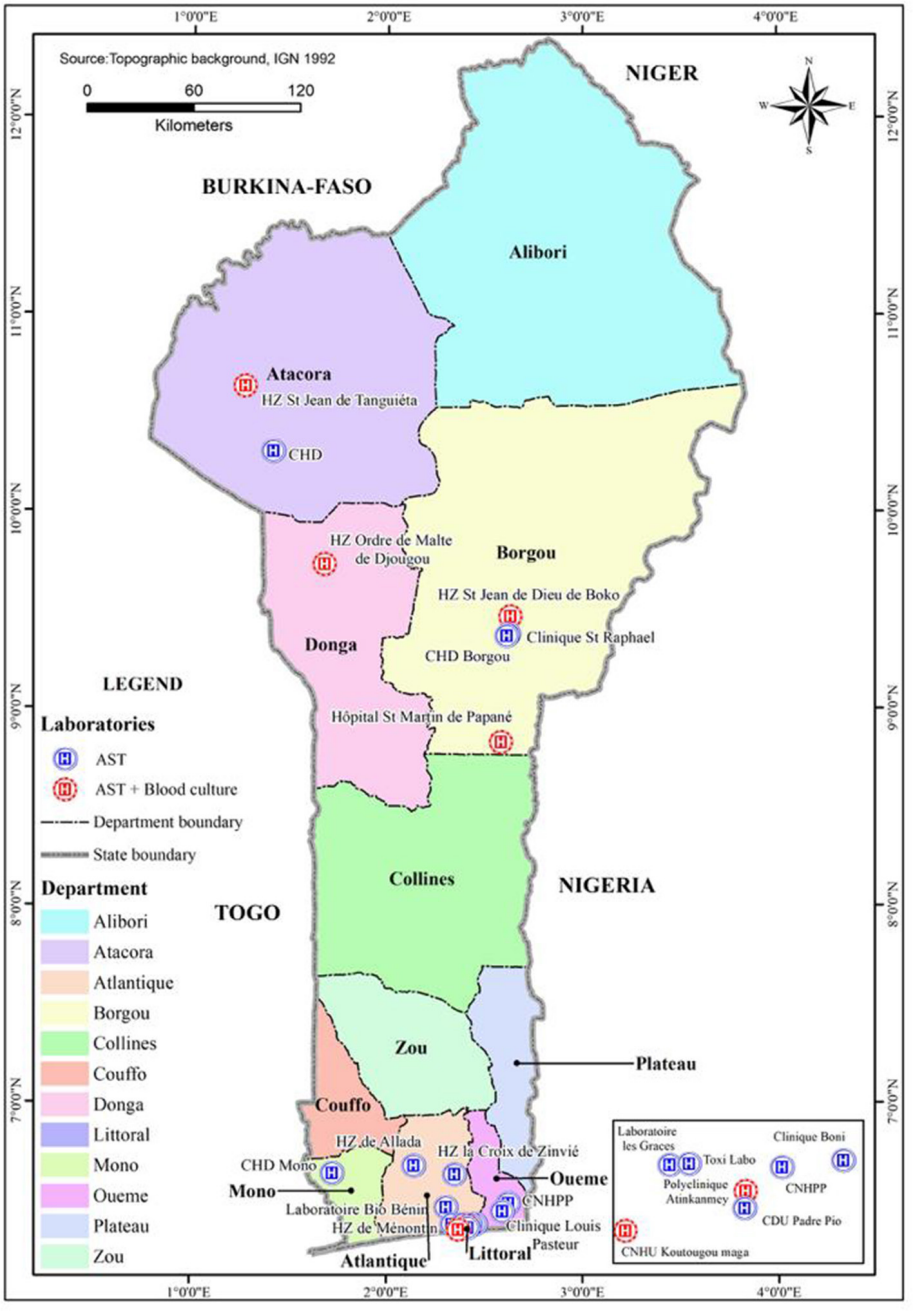
Distribution of the heathcare facilities with laboratories conducting blood cultures and/or antibiotic susceptibility testing (AST) in Benin. CNHPP, Centre National Hospitalier et Universitaire de Pneumo-phtisiologie; CNHU, Centre National Hospitalier Universitaire; CDU, Centre de Diagnostics et d'Urgences; CHD, Centre Hospitalier Départemental; HZ, Hôpital de Zone.

All 25 clinical bacteriology laboratories participating in the study conducted AST but only 6 of them also blood cultures (Figure 1). We inquired about the availability of certain basic resources of the laboratories to assess the general possibility of laboratories to do clinical microbiology work. Several laboratories lacked such basic resources as a microbiological safety cabinet, a freezer and internet connection (Supplementary Table S1). The background information of the interviewed staff showed that they all had a professional degree (Supplementary Table S2).

### Blood culture practice

The key data obtained from the 5 health care facilities and 1 independent laboratory that conduct blood cultures are shown in Table 1. In all the laboratories, the technicians indicated that they follow a standard blood sample processing procedure. They, for example, checked that the blood sample was taken correctly, appropriate bottles were used (aerobic blood culture bottles for adults or children), volume of blood was sufficient, and the weight in relation to the recommended average was acceptable. The recommended incubation temperature (35–37°C) was respected by all and the bottles grown in ordinary incubators were checked once, twice or more often per day for growth. Two laboratories used the BacT/ALERT automated system (BioMérieux, France), which signals when there is growth in the bottle. In the case of a positive blood culture, Gram staining was used to verify bacterial growth. The preliminary results were communicated to physicians to assist in early treatment.

**Table 1:** Practices related to a blood culture in the six laboratories that provide the test.

**Parameters**	**Number of laboratories (*N* = 6)**
**Person taking the blood culture sample**	
Nurse	6
Technician	4
Medical doctor	3
Trainee (Intern)	2
Not known (external samples)	3
**Blood culture vials used**	
Biomerieux	4
Liofilchem	2
**Average monthly number of blood cultures**	
Between 2 and 10	3
Between 11 and 20	1
20 or more	2
**Verification of proper blood sampling**	
Use of appropriate blood collection vials	6
Volume of blood collected	5
Attached patient information	4
Weighting the blood collection vials	1
**Incubation of blood culture bottles in**	
Ordinary microbiological incubator	4
Biomerieux automated system	2
**Incubation time of blood culture bottles**	
More than 5 days	4
4 days	1
1 day	1
**Incubation temperature of blood culture bottles**	
35–37°C	6
**Incubator temperature control procedure**	
Built-in thermometer	5
Separate thermometer in the incubator	1
**Frequency of checking the bottles in the incubator for growth**	
Once a day	1
Twice a day	2
More than twice a day	1
Machine signals in case of growth	2
**Indication for a positive blood culture**	
Uniform or subsurface turbidity	4
Gas production	3
Signal of the machine	2
Hemolysis	2
Flocculent deposit on the blood layer	2
White grains on surface or deep in the neck	1
Culturing after the predetermined time	1
Coagulation of the broth	0
A surface film	0
**Staining method for positive blood culture**	
Gram stain	6
**Agar plates used for positive blood cultures**	
Ready to use agar plates	2
Agar plates prepared in laboratory	2
Both	2
**Growth media for positive blood cultures**	
Blood agar	6
Chapman (biochemical identification media)	5
MacConkey	5
Chocolate	5
Eosin methylene blue agar	3
Sabouraud agar	2
Simmons citrate agar (biochemical identification media)	1
Bile esculin agar (biochemical identification media)	1
**Control procedure for media**	
Sterility test	6
Performance test	4
Visual observation	1
**Conservation of blood culture isolates**	
Yes	5
**Communication of blood culture results to a physician**	
In person	4
Phone	4
Multiple ways	3
Electronic transmission	1

Where growth in a blood culture bottle was detected, an aliquot was cultured on solid growth media (Table 1). For testing the quality of media, all the laboratories conducted a sterility test by incubating the media plates at 35–37°C for 24 h. However, only 4 conducted the performance test by growing some reference strains on the plates at 35–37°C for 24 h. According to the interviews, the most common bacteria isolated from blood cultures were: Gram-negative bacteria *Escherichia coli, Salmonella* spp*., Salmonella enterica* serovar Typhi and *Proteus mirabilis*, and gram-positive bacteria *Enterococcus* spp. and *Staphylococcus aureus* (Table 2).

**Table 2:** Most commonly isolated micro-organisms from the blood cultures in the six laboratories culturing blood.

**Laboratory**	**Most commonly isolated micro-organism**	**Second most commonly isolated micro-organism**	**Third most commonly isolated micro-organism**
1	*Salmonella* spp.	*P. mirabilis*	*S. aureus*
2	*Proteus* spp.	*E. coli*	*S. aureus*
3	*E. coli*	*Salmonella* Typhi	*S. aureus*
4	*E. coli*	*Staphylococcus* spp.	*Enterococcus* spp.
5	*S. aureus*	*E. coli*	*Streptococcus pneumoniae*
6	Yeast	*S. aureus*	*E. coli*

Three laboratories out of the six sometimes received samples from other hospitals. Yet, half of the laboratories processed a maximum of 10 blood samples a month. Limiting factors mentioned by the technicians and the laboratory managers for the low sample numbers were: availability and cost of the culture media (all laboratories used commercial blood culture vials) and other reagents, the method used, the lack of an automated system, the limited level of competence of the laboratory personnel and physicians' lack of knowledge about the importance of early antibiotic therapy.

When asked about their needs concerning conducting blood cultures, the staff expressed their need for equipment and consumables such as an automated blood culture machine and blood culture bottles. The laboratories also desired continuous training of staff to reinforce and develop their skills in detection of difficult-to-cultivate bacteria, standardization of procedures and conducting AST.

### Antibiotic susceptibility testing practice

Most of the laboratories used the disc diffusion method for AST (Table 3). They mainly followed the Comité de l'antibiogramme de la Société Française de Microbiologie (CA-SFM) or the European Committee on Antimicrobial Susceptibility Testing (EUCAST) Standards. Questions in our questionnaire were designed to assess the compliancy with the standard and the quality of work. For example, Muller-Hinton culture medium poured into petri dishes must be 4 ± 0.5 mm thick as defined by the CA-SFM standard. This measure was respected by 23 of 25 laboratories. Only 21 laboratories used McFarland standards to determine the concentration of the inoculum, while the rest assessed it by eye. The antibiotics for AST were primarily (72%) selected on the basis of CA-SFM/EUCAST standards (Table 3). The other criteria mentioned for selecting the antibiotics to be tested were: identity of the strain to be tested (64%) and availability of antibiotics in the laboratory (36%). Only 18 (72%) of the laboratories performed quality control of the disks. The reasons given for not performing quality control were: lack of materials, reference strains and expertise.

**Table 3:** Practices related to antibiotic susceptibility testing (AST) in the 25 laboratories that provide the test.

**Parameters**	**Proportion of laboratories (*****N*** = **25)**	
	***n***	**%**
**Average monthly number of samples tested for antibiotic susceptibility**		
≤10	6	24
11–20	6	24
>20	13	52
**AST method available**		
Antibiotic disc diffusion	22	88
E-test	3	12
**Agar medium used for AST**		
Muller Hinton	24	96
Cled	1	4
Eosin Methylene Blue	1	4
**Thickness of the medium in the petri dish**		
4 mm	23	92
>4 mm	2	8
**Procedure for determining inoculum**		
By eye without a standard	21	84
By eye using Mc Farland standard	4	16
**Inoculum spreading techniques**		
Swabbing	18	72
Flooding	10	40
Automated Biomerieux VITEK^®^ Compact system	3	12
**Criteria for selecting the antibiotics**		
CA-SFM/EUCAST	18	72
Depends on the bacterium to be tested	16	64
Depends on availability of antibiotic discs in laboratory	9	36
Depends on availability of antibiotics in pharmacy	1	4
CLSI	1	4
**Number of antibiotic discs tested per strain**		
≤10	7	28
11–20	17	68
>20	1	4
**Procedure for depositing antibiotic discs on agar plates**		
Sterile forceps	23	92
Antibiotic disc dispenser	7	27
**Time from depositing of antibiotic discs to placing the agar plates into incubator**		
20 min	12	48
10 min	11	44
1 h	1	4
0	1	4
**Incubation time of the agar plates**		
18–24 h	25	100
**Tools used for measuring the diameters of the inhibition zones**		
Ruler	18	72
Vernier caliper	5	20
Ruler and caliper	2	8
**Standards for interpretation of the AST results**		
CA-SFM/EUCAST	15	60
CA-SFM/EUCAST and the disc manufacturer's instructions	5	20
Disc manufacturer's instructions	5	20
**Storage temperature of the antibiotic discs**		
Refrigerator	24	96
Ambient room temperature	3	12
Freezer	1	4
**Carrying out quality control of antibiotic discs**		
Yes	18	72
**Procedure for the disc quality control**		
Use of a reference bacterial strain	13	52
Use of bacterial reference strain and determination of MIC	3	12
Determination of MIC	2	8
**Reasons for not performing quality control**		
Lack of materials	3	12
No reference strains	2	8
Lack of know-how	2	8
**Skills improvement needed**		
Yes	17	68
No	8	32

CA-SFM, Comité d'Antibiogramme de la Société Française de Microbiologie; EUCAST, European Committee on antimicrobial susceptibility testing; CLSI, clinical and laboratory standards institute; MIC, minimum inhibitory concentration.

Although all the laboratories included in the study conducted AST, many of them did notably few tests, even <10 per month (Table 3). The factors that limited the number of tests included (i) limited demand; (ii) insufficient human resources; (iii) insufficiency or shortage of laboratory equipment and consumables; (iv) negative blood culture samples; and (v) prescription of antibiotics by the physicians without requesting for a laboratory test. Staff in 17 of the 25 laboratories expressed their need to have their capacity strengthened to conduct quality AST. Specifically, capacity building in bacterial identification techniques, interpretation of AST results, quality control and choosing antibiotic discs were mentioned.

### Physicians' prescription practice and utilization of laboratory results

To get a general understanding of the use of antibiotics to treat infections, we asked the physicians for all the reasons for prescribing antibiotics (Supplementary Table S4). Digestive tract infections (85.5%) were the most common infections treated with antibiotics, followed by ear, nose and throat infections and upper respiratory infections (82.3%). Only one case of sepsis was mentioned, probably because sepsis was not considered as primary diagnosis in most cases. Only 50% of the respondents stated that they used a protocol defined by their hospital for the prescription of antibiotics. Among the physicians, who did not use any protocol, 83.9% justified it by the hospital's lack of a protocol for antibiotic therapy. In cases where antibiotics were used as a first-line treatment without bacteriological testing results, the antibiotics most commonly prescribed were penicillins, such as penicillin G, amoxicillin and ampicillin, in 87.1% of cases, followed by cephalosporins, such as cefoxitin, ceftriaxone and ceftazidime, in 82.3% of cases (Supplementary Table S4).

In treating sepsis, nearly all of the physicians interviewed recognized that the identification of the pathogen should influence the antibiotic therapy. However, in practice, empirical treatment was used in most cases due to the lack of testing capacity (Table 4). Physicians' knowledge of various factors responsible for antibiotic resistance was inadequate (Table 4). 80% of them recognized the inappropriate choice of antibiotics as one of the probable causes of therapeutic failures in curing the bacterial infections. Prescribed antibiotics not purchased by the patient, non-compliance with treatment, superinfections, resistant bacteria and low product efficiency were also mentioned.

**Table 4:** Physicians' knowledge and practices in prescribing antibiotics.

**Knowledge and practices**	**Proportion of physicians** **(*****N*** = **62)**	
	***n***	**%**
**Knowledge of the concept of antibiotic resistance**		
***Knowledge of natural resistance***		
Yes	56	90.3
***Means of recognition of antibiotic resistance***		
Treatment failure and persistence of symptoms after normal duration of treatment	35	57.4
Results of AST	26	42.6
***Factors believed to be responsible for antibiotic resistance***		
Poor quality of antibiotics available	55	88.7
Self-medication	54	87.1
Misuse of antibiotics	42	67.7
Non-compliance with the dosage	37	59.7
Inadequate duration of the antibiotic therapy	26	41.9
***Criteria other than AST for the choice of antibiotics***		
General knowledge about effective antibiotics	51	82.3
Availability of the antibiotic in Benin	43	69.4
Previous experience with the effectiveness of the antibiotic	34	54.8
Purchasing power of the patient	31	50
Availability of the antibiotic in the region	17	27.4
Cost of the antibiotic	7	11.3
Recommendation of a colleague	4	6.5
**Prescription practices**		
***Identification of the bacterium influences antibiotic therapy***		
Yes	60	96.8
***Important criteria for the choice of antibiotics***		
Results of AST	55	88.7
Experience with the effectiveness of an antibiotic	39	62.9
Usually prescribed antibiotics	29	46.8
Bacterial species and infection site	1	1.6
Clinical condition of the patient	1	1.6
***Preference for generic or brand-name antibiotics***		
Generic and brand-name antibiotics	27	43.5
Brand-name antibiotics	23	37.1
Generic antibiotics	18	29
***Factors that influence the preference to prescribe an antibiotic***		
Cost of the product	42	67.7
Unavailability of brand-name antibiotics	38	61.3
Quality of generics	33	53.2
Prescription protocol of the healthcare center	13	21
Effectiveness of an antibiotic	2	6.5
Financial capacity of the patient	1	1.6
***Probable reasons for encountered antibiotic treatment failures***		
Inappropriate choice of an antibiotic	42	79.2
Non-compliance of the patient with treatment	38	71.7
Antibiotic not purchased by the patient	23	43.4
Superinfections	19	35.8
Resistant pathogens	4	7.5
Low antibiotic efficiency	1	1.6
***Consulting an infectious disease specialist for the therapeutic protocol***		
Yes	5	8.1
***Reason for not consulting an infectious disease specialist***		
No infectiologist in the health facility	45	80.4
Has the necessary knowledge	6	10.7
***Postgraduate training for antibiotic prescribing***		
Yes	16	25.8

Of the 62 physicians interviewed, 54.8% had received 1 to 5 patients suspected to have sepsis in the preceding 12 months, while 9.7% had received none (Table 5). The patients were of all ages, although most commonly children. According to the physicians, in determining the diagnosis of sepsis, clinical symptoms were most important (91.1%) followed by blood culture results (73.2%). Third generation cephalosporins, ceftriaxone (82.1%) and cefotaxime (46.4%), were the most commonly used antibiotics in the treatment of sepsis.

**Table 5:** Management of sepsis and types of antibiotics prescribed.

**Parameters**	**Proportion of physicians** **(*****N*** = **62)**	
	***n***	**%**
**Number of patients with suspected sepsis, encountered during the last 12 months**		
1–5	34	54.8
>5	22	35.5
None	6	9.7
**Profile of patients with sepsis**		
People 15 years of age or older	26	46.4
Children under 5 years of age	24	42.9
Children 5–14 years of age	22	39.3
Newborns	19	33.9
Pregnant women	9	16.1
Patients with comorbidities	8	14.3
**Method of diagnosing sepsis in a patient**		
Clinical symptoms	51	91.1
Blood culture	41	73.2
Catecholamine testing	1	1.8
Blood count	1	1.8
**Means of communication with the laboratory in case of blood culture and AST were requested**		
By phone	54	96.4
In person	23	41.1
By an intermediary person (caregiver, nurse, etc.)	13	23.2
By e-mail	1	1.8
**Types of antibiotics used in treatment of sepsis**		
Ceftriaxone	46	82.1
Cefotaxime	26	46.4
Ceftazidime	19	33.9
Imipenem/cilastatin	18	32.1
Meropenem	14	25
Cefepime	10	17.9
Ampicillin and sulbactam	8	14.3
Levofloxacin	7	12.5
Gentamycin	7	12.5
Clindamycin	6	10.7
Ciprofloxacin	3	5.4
Metronidazole	2	3.6
Piperacillin and tazobactam	1	1.8

### Dispensing of antibiotics by pharmacies

When the pharmacists were asked about antibiotics they provide for sepsis, they answered that the medical prescription does not include the indication and therefore they do not know which antibiotics are for sepsis. However, they made some general suggestions for improving antibiotic delivery practices for sepsis treatment. These were (i) including all the required information in the prescription, (ii) having only medical doctors prescribe antibiotics, (iii) having pharmacists check the correctness of the prescription before dispensing the antibiotic, (iv) better control mechanism for dispensing antibiotics on medical prescription, and (v) general awareness raising and training on antibiotics, especially for rural population.

## Discussion

All 25 laboratories covered by this study conducted AST, but only six conducted blood cultures. This illustrates the limited capacity for microbiological diagnostics of sepsis in Benin. In the conduct of a blood culture, automated incubation and growth monitoring devices have almost become a standard in high-income countries, whereas this is far from being the case in LMICs (19). Most of the laboratories involved in our study had only very basic microbiological equipment, but two laboratories used an automated system for blood culturing. This equipment was provided by foreign partners rather than by the Beninese government. However, not even these laboratories conducted anaerobic blood cultures. In general, the main reasons reported by our interviewees for not conducting any blood cultures were the lack of equipment and the high cost. The situation is similar in most LMICs which face many challenges in implementing blood cultures due to financial, logistical and infrastructural constraints (19). As a detail, it is worth mentioning that only 40% of the laboratories surveyed had a microbiological safety cabinet. This compromises the safety of the staff as well as the quality of work (20).

Even in the six laboratories conducting blood cultures, the number of samples processed was very low. Furthermore, the availability of microbiological testing in Benin is geographically very biased. In 8 out of the 12 departments there is no laboratory conducting a blood culture, none in the whole central Benin. For patients this means, according to our discussions with the laboratory staff, that an accompanying person is obliged to travel several hundred kilometers to collect blood culture bottles from Cotonou, have the patient's sample taken in a treating hospital and take the bottle back to Cotonou, where two of the main laboratories conducting blood cultures are located. They might have to make the same round trip to get the results. In the north-west, the healthcare facilities without possibility for blood culture may forward the patient to another facility. For example, at the Atacora CHD, they reported that the lack of equipment and consumables for blood cultures leads the hospital to transfer patients to the hospitals in Djougou or Tanguiéta, which are located in a distance of about an hour's drive. The latter hospitals are supported by the Catholic Church (Order of Malta Hospital in Djougou and St Jean de Dieu Hospital in Tanguieta) and have better laboratories than the governmental hospitals, due to better funding. In general, the public hospitals are seriously underfunded, and consequently most of the laboratories conducting blood cultures and AST are either private or faith-based, as shown by our study.

Besides the poor access to the bacteriological diagnostics, the quality of laboratory results is a problem. In our questionnaires, we had many questions related to the quality of testing and quality control practices, since erroneous results can lead to inappropriate treatment of a patient. The laboratory staff indicated that they follow a standard in processing blood samples in the laboratory. For example, they controlled the volume of blood collected, since the sensitivity of blood culture depends on the volume. Blood culture bottles were incubated typically for 5 days and checked daily for bacterial growth. In literature, some authors recommend blind sub-culturing within the first 24 h of incubation as an effective strategy for rapid detection but the recent study in Benin did not recommend it because of increased work load and risk of contamination (14). Regardless of the good intentions of the laboratory staff, our survey showed that there is discrepancy between the standard procedure and daily practice.

The major deficiency reported by the laboratory staff themselves was related to identification of bacteria in case of a positive blood culture. They reported *Salmonella* spp., *E. coli, S. enterica* serovar Typhi*, Enterococcus* spp. and *Staphylococcus aureus* to be the most commonly isolated bacteria from blood cultures. These findings partly match the results of the recent study in Benin, indicating *Klebsiella pneumoniae, S. enterica* serovar Typhi, *S. aureus, E. coli, Enterobacter cloaceae* and non-typhoidal *Salmonella* spp. as the most common isolates from sepsis in a Boko district hospital in central Benin (14). In another study, the most common bacteria isolated from neonatal sepsis cases in Africa were *K. pneumoniae, Klebsiella michiganensis, S. aureus, Serratia marcescens* and *Burkholderia cepacia* (4). However, the laboratories interviewed did not seem to be able to identify *Klebsiella* consistently, not even in the hospital involved in the study of Ombelet et al. (14), since none of them mentioned *Klebsiella* among the three most common isolates from the blood cultures. This is possibly due to the difficulty in distinguishing between *Klebsiella* and *E. coli*. Also earlier observations on identification of *K. pneumoniae* have indicated limited accuracy in many LMICs (21). However, *K. pneumoniae* might indeed be one of the most common causes of sepsis in Africa, with potentially high virulence and multidrug resistance properties (22). Therefore, its epidemiological surveillance should be a priority. One of the laboratories included in our study reported yeast as the most commonly isolated microorganism from blood cultures. Indeed, yeasts are among the microorganisms isolated from bloodstream infections, but they are a relatively rare finding (4, 14, 23, 24). It can therefore be assumed that, given the very low number of positive blood cultures in the laboratory in question, contamination and misidentification distorted the results. All the technicians we interviewed were particularly interested in improving their knowledge and practices in bacterial identification methods.

Our results showed that the physicians recognized the importance of a blood culture in the diagnosis of sepsis, but in practice, the rate of testing was very low. There appears to be a vicious circle, where physicians do not request laboratory tests due to their non-availability, patient's inability to pay for them, slow processing and unreliability of the results, and the low demand for laboratory tests leads to poor resourcing of laboratories. Consequently, physicians prescribe antibiotics on a probabilistic basis. Furthermore, hospitals have no standardized guidelines to support prescription practice. Only 5 of the physicians interviewed reported consulting infectious diseases specialists when prescribing antibiotics. 45 specifically mentioned the absence of infectious disease specialists from their health facility.

According to our survey, factors limiting testing included insufficient properly trained personnel, insufficient or broken laboratory equipment and lack of consumables. The analysis of practices related to AST revealed technical deficiencies concerning the choice of antibiotic discs and the quality control of the discs, media and growth of reference bacteria. Only 18 (72%) of the laboratories performed quality control of the discs although it is strongly recommend by the EUCAST standards to ensure that efficacy has not been recuded by e.g., poor storage or other conditions. The antibiotics chosen for AST were sometimes chosen based on availability of antibiotics in the pharmacies near the hospital. This might be rational considering the treatment but does not provide proper surveillance information for the local situation. The majority of technicians expressed their need and willingness for further training in AST. It was also seen to be important to standardize the practices at the national level to improve the quality of microbiological testing.

In Benin it is a common, but unofficial, practice that nurses rather than medical doctors prescribe antibiotics (25), which the pharmacists indicated as a major problem due to the insufficient training of nurses. Thus, in future studies exploring the prescription of antibiotics and in training programs, it would be important to include nurses as well as physicians. Furthermore, in West Africa, many antibiotics are sold in pharmacies without prescription (only one pharmacy in our study admitted this) and by street vendors (26). The different galenic forms can also lead to confusion among prescribers and patients in the correct use of the antibiotics (27). Therefore, optimizing the monitoring of antibiotic delivery is also a way to improve antibiotic use practices (28). Moreover, the quality of antibiotics and many other medicines in LMICs is often substandard (27). One factor that contributes to a partial or total reduction in the quality of antibiotics in the hot and humid climate in West Africa is poor storage (29). This issue was also mentioned in our interviews. Proper storage of antibiotics is costly and requires well trained personnel, which is in short supply in West African countries. Although it is well-known that poor quality of antibiotics leads to an increase in multi-resistant bacteria and the risk of therapeutic failure, very few LMICs have a quality control agency to monitor the quality of medicines (30, 31).

Our main reason for undertaking this study was to gain understanding of the base-line level of the bacteriological laboratory diagnostics of blood culture and AST for bacteria that cause sepsis. We utilized the results in designing a training module for laboratory technicians to improve their competence (details will be reported elsewhere). The laboratory staff that participated in the study appreciated our effort to contact all laboratories across the country and address their concerns. We are planning a follow-up training course concentrating on identification of key bacteria causing sepsis. Since successful treatment and prevention of infections requires multi-professional collaboration, we also interviewed physicians and pharmacists on their knowledge of sepsis and usage of antibiotics. Based on the obtained results, we have organized events to bring the different professional groups and the national health authorities together to discuss the best practices in the local settings.

In conclusion, we recognize an urgent need to increase the availability and quality of blood cultures and AST for improved sepsis management throughout Benin. Laboratories with a clinical bacteriology unit must be provided with appropriate equipment and more consumables to ensure that there is at least one laboratory able to conduct necessary diagnostics in each of Benin's 12 departments. The laboratory staff involved in this study themselves expressed their need and willingness to strengthen their skills in conducting both blood cultures and AST. Also reinforced collaboration between the laboratories, physicians and pharmacists is necessary for improved sepsis management.

## Data availability statement

The original contributions presented in the study are included in the article/Supplementary material, further inquiries can be directed to the corresponding author.

## Author contributions

BL, VD, HK, SM, RE, MP, HB, and KH designed the study. BL, VD, and HK collected and analyzed the data. BL wrote and VD, HK, SM, RE, MP, HB, and KH revised the manuscript. All authors contributed to the article and approved the submitted version.
