# Supplementary material for: Assessment of blood cultures and antibiotic susceptibility testing for bacterial sepsis diagnosis and utilization of results by clinicians in Benin: A qualitative study
Source: Front Public Health. 2023 Jan 16;10:1088590. doi: 10.3389/fpubh.2022.1088590 (PMC9885088; doi:10.3389/fpubh.2022.1088590)
Supplement: Supplementary file 1 [file Data_Sheet_1.docx]

**Table S1.** Resources available in the 25 clinical bacteriological laboratories

| Resources available | Number of laboratories (N=25) |
| --- | --- |
| Microscope | 25 |
| Microscope with 100x objective | 25 |
| Refrigerator | 25 |
| Incubator | 25 |
| Ready-to-use powder for preparation of selective media | 25 |
| Autoclave | 24 |
| Stable source of electricity | 24 |
| Computer | 23 |
| Distillation or filtering device for purified water | 19 |
| Blood agar made from human blood | 15 |
| Freezer | 14 |
| Internet | 14 |
| Microbiological safety cabinet | 10 |
| Agar diffusion tests (E-tests) | 5 |

**Table S2.** Socio-demographic characteristics of the enrolled participants

| Socio-demographic characteristics | Laboratory technicians (N=25) | Laboratory managers (N=25) | Physicians (N=62) | Pharmacists (N=47) |
| --- | --- | --- | --- | --- |
|  | n | n | n | n |
| Gender |  |  |  |  |
| Female | 8 | 7 | 21 | 25 |
| Male | 17 | 18 | 41 | 23 |
| Age (in years) | | | | |
| Under 30 | 5 | 0 | 13 | 18 |
| 0 or older | 20 | 25 | 49 | 30 |
| Length of time in the profession (in years) | | | | |
| Less than 5 | 12 | 8 | 27 | 21 |
| 5 to 9 | 6 | 6 | 21 | 17 |
| 10 to 14 | 2 | 3 | 9 | 5 |
| 15 to 19 | 3 | 5 | 3 | 4 |
| 20 or more | 2 | 3 | 2 | 1 |
| Professional degree |  |  |  |  |
| MSc (Microbiologist) | 5 | - | 0 | 0 |
| BSc (Biomedical Analysist) | 17 | - | 0 | 0 |
| DIT (Biomedical Analysist) | 3 | - | 0 | 0 |
| MD (General practitioner) | 0 | - | 38 | 0 |
| MD (Gynecologist) | 0 | - | 6 | 0 |
| MD (Pediatrician) | 0 | - | 13 | 0 |
| MD (Surgeon) | 0 | - | 3 | 0 |
| MSc (Anesthesiologist) | 0 | - | 2 | 0 |
| PD (Pharmacist) | 0 | - | 0 | 34 |
| PD (Pharmacy assistant) | 0 | - | 0 | 9 |
| BSc (Nurse) | 0 | - | 0 | 5 |

BSc: Bachelor in Science; DIT: Diplome d’ingenieur des Travaux; MD: Medical Doctor; MSc: Master of Science; PD: Pharmacist Doctor. The degree held by the laboratory managers was not recorded (-).

**Table S3**: Antibiotic families used for antibiotic susceptibility testing

| Antibiotic family | Number of laboratories (N=25) |
| --- | --- |
| Aminoglycosides | 22 |
| Amoxicillin/Clavulanic Acid ^a^ | 1 |
| Betalactams ^a^ | 10 |
| Carbapenems ^a^ | 11 |
| Cephalosporins ^a^ | 14 |
| Chloramphenicol | 6 |
| Fluoroquinolones and quinolones | 23 |
| Fosfomycine | 2 |
| Glycopeptides | 2 |
| Lincosamides | 2 |
| Macrolides | 16 |
| Monobactams ^a^ | 6 |
| Nitrofurane | 3 |
| Penicillins ^a^ | 11 |
| Polymyxins | 3 |
| Rifampicin | 1 |
| Sulfonamides | 3 |
| Tetracyclines | 7 |

^a^Answers were given to the open question ‘’Which families of antibiotics do you use for AST?’’. Some laboratories answered “Betalactams” whereas others mentioned the subgroups.

**Table S4:** Reasons for prescribing and choice of antibiotics in bacterial infections

| Parameters | Proportion of physicians (N=62) | |
| --- | --- | --- |
|  | n | % |
| Indications for prescribing antibiotics during the last 12 months | | |
| Digestive tract infections | 53 | 85.5 |
| Ear, nose and throat infections and upper respiratory infections | 51 | 82.3 |
| Skin infections | 45 | 72.6 |
| Lower respiratory infections | 44 | 71.0 |
| Urogenital infections | 43 | 69.4 |
| Cerebro-meningeal infections | 30 | 48.4 |
| Osteoarticular infections | 26 | 41.9 |
| Ocular infections | 9 | 14.5 |
| Sepsis | 1 | 1.6 |
| Intraoperative antibiotic prophylaxis | 1 | 1.6 |
| Chosen therapy when suspecting a bacterial infection | | |
| Antibiotic therapy followed by bacteriological examination | 42 | 67.7 |
| Treatments other than antibiotics and request for laboratory diagnosis | 27 | 43.5 |
| Empirical antibiotic therapy | 9 | 14.5 |
| Use of a hospital protocol for antibiotic therapy | | |
| Yes | 31 | 50,0 |
| Reason for not using a protocol for antibiotic therapy | | |
| No protocol available in the hospital | 26 | 83.87 |
| Waiting for the AST results | 2 | 6.45 |
| Antibiotic families often used as first-line treatment without bacteriological examination results | | |
| Penicillins | 54 | 87.1 |
| Cephalosporins | 51 | 82.3 |
| Aminoglycosides | 29 | 46.8 |
| Macrolides and related products | 26 | 42.9 |
| Fluoroquinolones | 24 | 38.7 |
| Carbapenems | 11 | 17.7 |
| Monobactams | 3 | 4.8 |
| Glycopeptides | 3 | 4.8 |
| Sulfonamides | 3 | 4.8 |
| Polymyxins | 2 | 3.2 |
| Rifamycins | 1 | 1.6 |
| Tetracyclines | 1 | 1.6 |
